# Clinical Characteristics and Treatment Outcomes of *Clostridium difficile* Infections by PCR Ribotype 017 and 018 Strains

**DOI:** 10.1371/journal.pone.0168849

**Published:** 2016-12-21

**Authors:** Jieun Kim, Yeonjae Kim, Hyunjoo Pai

**Affiliations:** 1 Department of Internal Medicine, College of Medicine, Hanyang University, Seoul, Korea; 2 Center for Infectious Diseases, National Medical Center, Seoul, Korea; Institut Pasteur, FRANCE

## Abstract

The objective of this study was to identify the clinical characteristics of *Clostridium difficile* infections (CDIs) caused by toxin A-negative/toxin B-positive (A-B+) PCR ribotype 017 (R017) and A+B+ ribotype 018 (R018) strains, prevalent in Asian countries. From February 2010 through January 2013, all CDI patients in our hospital were enrolled; their medical records were retrospectively reviewed, and the isolates were characterized by toxigenic culture and PCR ribotyping. Based on the ribotypes, a total of 510 cases were categorized as R017 (139, 27.3%), R018 (157, 30.8%) and other ribotypes groups (214, 42.0%), and clinical variables were compared between R017 and other ribotypes, R018 and other ribotypes and R018 and R017 groups. The patients with R017 infections had a higher mean Charlson’s comorbidity index (OR 1.1, 1–1.21, *p* = 0.05), lower serum albumin (OR 0.47, 0.31–0.73, *p* = 0.001) and lower CRP levels (OR 0.96, 0.92–0.99, *p* = 0.022) than those with other ribotypes. R018 infections caused more azotemia (OR 4.06, 1.28–12.91, *p* = 0.018) and more frequent severe CDI (OR 1.87, 1.12–3.13, *p* = 0.016) than the other ribotypes infections. R017 and R018 infections were more often associated with toxin positive stools (OR 2.94, 1.65–4.09, *p*<0.001; OR 4.55, 2.82–7.33, *p*<0.001). In terms of treatment outcomes, R017 infections caused a marginally higher 30-day mortality than other ribotypes infection. In a final multiple logistic regression model, 30-day mortality was associated with leukocytosis (OR 2.45, 1.0–6.01, *p* = 0.05) and hypoalbuminemia (OR 4.57, 1.83–11.39, *p* = 0.001), but only marginally with R017 infection (OR 2.14, 0.88–5.22, *p* = 0.094). In conclusion, infections by *C*. *difficile* R018 strains tend to cause more severe disease, while there was a trend for higher mortality with R017 infections.

## Introduction

*Clostridium difficile* causes symptoms from mild diarrhea to pseudomembranous colitis (PMC), mostly in elderly people who have been exposed to antibiotics. The increased incidence and severity of *C*. *difficile* infections (CDI) due to an epidemic of a BI/NAP1/PCR ribotype 027 strain in North America are well documented [1]. In Asian countries, ribotype 027 strains have caused sporadic cases of CDI, but the most prevalent ribotypes are 017, 018, 014, 002 and 001 [2–5].

Variant toxin A-negative/toxin B-positive strains of PCR ribotype 017 (R017) are widespread in Asian countries such as Korea, China and Japan and have caused epidemics worldwide [2, 4–6]. Previous clindamycin use and young age were associated with R017 infections in Korea [2], and antineoplastic agents, use of nasal feeding tubes, and care in a particular hospital ward were risk factors in Japan [7]. R017 is among several clonal lineages of *C*. *difficile* known to cause parallel increases in disease severity, mortality and recurrence [8, 9], but having similar clinical outcome in a report [2].

Toxin A-positive/toxin B-positive PCR ribotype 018 (R018) strains are the most common ribotype in Japan, and have become the most prevalent strains isolated in Korea [6, 10, 11]. R018 strains are also prevalent in Italy [12, 13], and were the 4^th^ most common strain in Europe in 2008 [14]. R018 strains are successful strains which survive well in hospital environments because they are highly resistant to several antibiotics [10, 12, 13]. However the clinical characteristics of R018 infections are not yet well established.

The objective of this study was to clarify the clinical characteristics and treatment outcomes of CDI caused by the R017 and R018 strains prevalent in Asian countries compared with strains of other ribotypes.

## Patients and Methods

### Patients and study design

From February 2010 through January 2013, all patients confirmed to have healthcare-associated *C*. *difficile* infections in Hanyang University Hospital, a 900-bed tertiary care facility in Seoul, South Korea were enrolled. The study was approved by the Institutional Review Board of Hanyang University Hospital (HYUH IRB 2012-07-023). Informed consent was waived by the Board.

### Definitions

Diarrhea was defined as unformed stools ≥ 3 times per day on two consecutive days, and CDI was confirmed when toxigenic culture was positive in diarrheal patients, or the toxin assay A&B (VIDAS^®^*C*. *difficile* Toxin A & B; BioMerieux SA, Marcy l’Etoile, France) yielded positive results, and/or pseudomembranes were seen by endoscopy or histology [10]. Healthcare-associated CDI (HA-CDI) was diagnosed in patients who developed diarrhea at least 72 hour after hospitalization or within two months of their last discharge from hospital [15].

Clinical cure was defined as resolution of diarrhea within the treatment period. This required conversion to no more than two semi-formed or formed stools per day [15]. Recurrence was defined as growth of *C*. *difficile* with toxin genes, positive toxin assay A&B, or pseudomembranes, with recurrence of symptoms between the end of treatment and 30 days later.

### Data collection

Medical records were reviewed retrospectively. Age, sex, length of hospital stay, history of admissions within the previous two months, and underlying disease including Charlson’s score were collected as demographic data [16]. Charlson’s score is composed with 17 comorbid conditions known to be associated with 1-year mortality such as myocardial infarction, congestive heart failure, cerebrovascular diseases, diabetes and more, and to measure disease burden, a weighted score to each comorbid condition based on the relative risk of 1-year mortality is used. Use of antibiotics and proton pump inhibitors within the previous two months was also recorded. Disease severity of CDI was assessed through two methods. First, the sum of scores ascribed to the following factor (1 point each) was measured: age over 60 years, temperature over 38.3°C, albumin level below 2.5 mg/dL, or WBC count ≥ 15,000 cells/μL. When the score was ≥ 2, disease was regarded as severe [8]. Second, if a patient had a WBC count ≥ 15,000 cells/μL or a>1.5 fold rise in serum creatinine, the disease was regarded as severe [17].

### Isolation of *C*. *difficile* and microbiological characterization

Stool specimens were cultured anaerobically on cycloserine–cefoxitin–taurocholate agar (Oxoid Ltd., Cambridge, UK) supplemented with 7% horse blood, after alcohol shock treatment. Colonies of *C*. *difficile* were identified with an API® Rapid ID 32A system (bioMérieux SA, Lyon, France). Multiplex PCR and agar gel electrophoresis were performed with DNA from the cultured *C*. *difficile* isolates, as described elsewhere with minor modifications [8, 10]. PCR-ribotyping was performed as described elsewhere [8, 10], and banding patterns were checked by eye. Each unique pattern was assigned its own ribotype code and was matched with the PCR-ribotypes of reference strains ribotype 027 (BI/NAP1/027), ribotype 017 (ATCC 43598) and standard strains from the ECDC-Brazier collection.

### Statistical methods

SPSS version 18.0 for Windows (SPSS Inc., Chicago, IL, USA) was used for statistical analysis. Spearman’s rank correlation analysis or the chi-square test was performed as appropriate. A *p*-value <0.05 in a two-tailed test was considered statistically significant.

## Results

During the study period, from February 2010 through January 2013, the incidence of CDI was 80.4/100,000 patient days, and a total of 510 HA-CDI patients including 81 recurred cases (15.9%) were enrolled. R018 (157 isolates, 30.8%) and R017 (139 isolates, 27.3%) were the most prevalent ribotypes. All isolates of R017 were toxin A-negative, toxin B-positive and CDT-negative, and R018 isolates were toxin A-positive, toxin B-positive and CDT-negative. Other ribotypes identified in this study except R017 and R018 were R001, R015, R112, R014, R293, R012, R002, R078, R163, R106, R267 in order of frequency, and 131 strains were undefined ribotypes.

Thus the 510 patients were categorized as R017 group (n = 139), R018 group (n = 157) and an “other ribotypes” group (n = 214). In order to identify the specific clinical characteristics and treatment outcomes of CDIs caused by R017 and R018 isolates, variables were compared between the R017 and other ribotypes groups, R018 and other ribotypes groups and finally the R018 and R017 groups, respectively.

### Demographics and medical histories of the CDI patients

Table 1 presents the demographics and medical histories of the three groups. Compared to other ribotypes group, the R017 group had a higher Charlson’s comorbidity index, stayed longer in hospital, and had used 2-fold more clindamycin or carbapenem, respectively, and 10-fold more rifampin within the past 2 months in multivariate analysis. The R018 group had fewer differences in demographics and medical histories from other ribotypes group; but it had a higher proportion of elderly patients, and had received more metronidazole and proton pump inhibitor (PPI) within the previous 2 months. In comparison between R018 and R017 groups, R018 group contain more female patients and shorter duration of hospitalization, but had used more 1^st^ generation cephalosporin, metronidazole and PPI during 2 months.

**Table 1:** Demographics and medical histories of *Clostridium difficile* infections with ribotypes 017 (R017) and 018 (R018)

	No. (%) of patients (N = 510)			ribotype 017 vs. others				ribotype 018 vs. others				ribotype 018 vs. ribotype 017			
	ribotype 017 (N = 139)	ribotype 018 (N = 157)	others (N = 214)	simple OR	*p* value	multiple OR	*p* value	simple OR	*p* value	multiple OR	*p* value	simple OR	*p* value	multiple OR	*p* value
**Sex (male) N (%)**	74 (53.2)	58 (36.9)	90 (42.1)	1.57 (1.02, 2.41)	0.04	1.03 (0.63, 1.70)	0.895	0.81 (0.53, 1.23)	0.321			0.52 (0.32, 0.82)	0.005	0.54 (0.33, 0.88)	0.014
**Age (years) median (1Q, 3Q)**	72 (59,77)	72 (64,78)	67 (56,76)	1.02 (1.00, 1.03)	0.039	1.00 (0.99, 1.02)	0.695	1.02 (1.00, 1.03)	0.015	1.02 (1.00, 1.03)	0.039	1.00 (0.99, 1.02)	0.72		
Charlson comorbidity index median(1Q, 3Q)	3 (1,6)	3 (1,4.5)	3 (1,5)	1.11 (1.02, 1.2)	0.017	1.1 (1, 1.21)	0.05	1.03 (0.94, 1.12)	0.558			0.91 (0.83, 1.00)	0.061		
**Hospital days before inclusion median (1Q, 3Q)**	25 (11,59)	15 (6.5, 41)	14.5 (5, 36.3)	1.01 (1.00, 1.01)	0.001	1.01 (1.00, 1.02)	0.001	1.00 (1.00, 1.01)	0.309			1.00 (0.99, 1)	0.034	0.99 (0.99, 1.00)	0.005
History of admission within 2 months	55 (39.6)	59 (37.6)	71 (33.2)	1.32 (0.85, 2.05)	0.221			1.21 (0.79, 1.87)	0.38			0.92 (0.58, 1.47)	0.726		
Antibiotic use within 2 months	137 (98.6)	151 (96.2)	190 (88.8)	8.65 (2.01, 37.23)	0.004			3.18 (1.27, 7.98)	0.014			0.37 (0.07, 1.85)	0.225		
** 1st generation cephalosporins**	18 (12.9)	35 (22.3)	41 (19.2)	0.63 (0.34, 1.15)	0.129			1.21 (0.73, 2.01)	0.46			1.93 (1.04, 3.59)	0.038	1.94 (1.01, 3.76)	0.048
** **2nd generation cephalosporins	25 (18.0)	32 (20.4)	37 (17.3)	1.05 (0.6, 1.84)	0.867			1.23 (0.72, 2.07)	0.45			1.17 (0.65, 2.09)	0.602		
** **3rd generation cephalosporins	52 (37.4)	70 (44.6)	85 (39.7)	0.91 (0.59, 1.41)	0.664			1.22 (0.81, 1.85)	0.348			1.35 (0.85, 2.15)	0.211		
** **4th generation cephalosporins	24 (17.3)	19 (12.1)	18 (8.4)	2.27 (1.18, 4.37)	0.014	1.14 (0.53, 2.48)	0.741	1.50 (0.76, 2.96)	0.243			0.66 (0.34, 1.27)	0.21		
** **BL/BLI	44 (31.7)	47 (29.9)	65 (30.4)	1.06 (0.67, 1.68)	0.799			0.98 (0.63, 1.53)	0.928			0.92 (0.56, 1.51)	0.749		
** **Glycopeptide	38 (27.3)	35 (22.3)	38 (17.8)	1.74 (1.04, 2.91)	0.033	0.90 (0.48, 1.68)	0.734	1.33 (0.80, 2.22)	0.278			0.76 (0.45, 1.30)	0.316		
** **2nd quinolones^a^	35 (25.2)	34 (21.7)	45 (21.0)	1.26 (0.76, 2.09)	0.363			1.04 (0.63, 1.72)	0.884			0.82 (0.48, 1.41)	0.475		
** **3rd quinolones^b^	50 (36.0)	38 (24.2)	37 (17.3)	2.69 (1.64, 4.41)	<0.001	1.76 (0.92, 3.36)	0.089	1.53 (0.92, 2.54)	0.103			0.57 (0.64, 0.94)	0.028	0.82 (0.43, 1.56)	0.544
** **Aminoglycosides	17 (12.2)	17 (0.8)	18 (8.4)	1.52 (0.75, 3.06)	0.243			1.32 (0.66, 2.66)	0.432			0.87 (0.43, 1.78)	0.706		
** Clindamycin**	33 (23.7)	23 (14.6)	19 (8.9)	3.20 (1.73, 5.89)	<0.001	2.36 (1.07, 5.21)	0.034	1.76 (0.92, 3.36)	0.086			0.55 (0.31, 1.00)	0.048	0.71 (0.33, 1.51)	0.371
** Metronidazole**	29 (20.9)	49 (31.2)	38 (17.8)	1.22 (0.71, 2.09)	0.468			2.10 (1.29, 3.42)	0.003	1.73 (1.04, 2.87)	0.035	1.72 (1.01, 2.93)	0.045	1.81 (1.03, 3.21)	0.041
** **Carbapenem	28 (20.1)	22 (14.0)	18 (8.4)	2.75 (1.45, 5.19)	0.002	2.26 (1.09, 4.67)	0.028	1.77 (0.92, 3.44)	0.089			0.65 (0.35, 1.19)	0.162		
** **Macrolide	7 (5.0)	7 (4.5)	6 (2.8)	1.84 (0.61, 5.59)	0.283			1.62 (0.53, 4.91)	0.396			0.88 (0.30, 2.57)	0.815		
** Rifampin**	22 (15.8)	0 (0.0)	5 (2.3)	7.86 (2.9, 21.30)	<0.001	10.00 (3.47, 28.80)	<0.001		0.076*				<0.001*		
Steroid	41 (29.5)	53 (33.8)	54 (25.2)	1.24 (0.77, 2.00)	0.378			1.51 (0.96, 2.37)	0.074			1.22 (0.75, 1.99)	0.432		
Chemotherapy	19 (13.7)	22 (14.0)	22 (10.3)	1.38 (0.72, 2.66)	0.333			1.42 (0.76, 2.67)	0.274			1.03 (0.53, 1.99)	0.932		
Immunosuppresants	6 (4.3)	5 (3.2)	12 (5.6)	0.76 (0.28, 2.07)	0.591			0.55 (0.19, 1.61)	0.276			0.73 (0.22, 2.44)	0.609		
**PPI use**	49 (35.3)	73 (46.5)	71 (33.2)	1.10 (0.7, 1.72)	0.688			1.75 (1.15, 2.67)	0.01	1.61 (1.04, 2.49)	0.034	1.60 (1.00, 2.55)	0.05	1.88 (1.12, 3.16)	0.017

* p value by Pearson’s chi-square test or Fisher’s exact test

BL/BLI, β-lactam/β-lactamase inhibitor; PPI, proton pump inhibitor

^a^2nd quinolones include ciprofloxacin, ofloxacin, norfloxacin, lomefloxacin and enoxacin, but mostly ciprofloxacin in this study.

^b^3rd quinolones include levofloxacin, sparfloxacin, gatifloxacin and moxifloxacin.

### Clinical characteristics of CDI patients

Table 2 presents the clinical characteristics of the R017, R018 and other ribotypes groups. The R017 group differed in a couple of clinical characteristics from other ribotypes group; it had lower albumin (OR 0.47, 0.313–0.73, *p* = 0.001) and CRP (OR 0.96, 0.92–0.99, *p* = 0.022) levels but higher positive EIA toxin assay (OR 2.59, 1/65-4.09, *p*<0.001). On the other hand, R018 infections differed from the other ribotype infections in many ways in univariate analysis; they occurred more in elderly patients and caused more leukocytosis, and more frequent azotemia; they therefore resulted in a higher severity index by the 2 methods of assessing severity. Also the result of toxin assays by ELISA tended to be more often positive in R018 infections. In terms of treatment, more patients in R018 and R017 groups received specific treatment against *C*. *difficile*; three quarters of the R018 and 68% of the R017 but a half of patients with other ribotypes were treated for CDI. However, metronidazole was common antibiotics used in 9 of 10 patients among all 3 groups. In a multivariate analysis, R018 infections were associated with more frequent azotemia (OR 4.06, 1.28–12.91, *p* = 0.018) and severe CDI (OR 1.87, 1.12–3.13, *p* = 0.016) assessed by the factors of age > 60 years, temperature > 38.3°C, albumin level < 2.5 mg/dL, or WBC count ≥ 15,000 cells/μL, and they were much more likely to be toxin positive than the other ribotype infections (OR 4.55, 2.82–7.33, *p*<0.001). In a multivariate analysis comparing between R018 and R017 infections, R018 infections caused a higher CRP level (1.04, 1.01–1.08, *p* = 0.021), less frequent hypoalbuminemia (0.41, 0.2–0.84, *p* = 0.014), but more frequent severe CDI assessed by leukocytosis and azotemia (1.88, 1.05–3.37, *p* = 0.034).

**Table 2:** Clinical characteristics and treatment outcomes of *Clostridium difficile* infections with ribotypes 017 (R017) and 018 (R018)

	total number = 510			ribotype 017 vs. others				ribotype 018 vs. others				ribotype 018 vs. ribotype 017			
Characteristics	ribotype 017 (N = 139)	ribotype 018 (N = 157)	others (N = 214)	simple OR	*p* value	multiple OR	*p* value	simple OR	*p* value	multiple OR	*p* value	simple OR	*p* value	multiple OR	*p* value
CDI episode															
1st CDI	117 (84.2)	127 (80.9)	185 (86.4)												
Recurred CDI	22 (15.8)	30 (19.1)	29 (13.6)	1.2 (0.66, 2.19)	0.553			1.51 (0.86, 2.63)	0.15			1.26 (0.69, 2.3)	0.46		
Laboratory findings median (1Q, 3Q)															
WBC (*10^3^/mm^3^)	8000 (6300,13000)	9800 (7200,15400)	8650 (900,12325)	1.00 (1.00, 1.00)	0.21			1.00 (1.00, 1.00)	0.007			1.00 (1.00, 1.00)	0.258		
**Albumin (mg/dL)**	3.0 (2.6,3.4)	3.0 (2.7,3.5)	3.2 (2.8,3.5)	0.57 (0.38, 0.84)	0.004	0.47 (0.31, 0.73)	0.001	0.78 (0.53, 1.15)	0.207			1.41 (0.94, 2.13)	0.097		
**CRP**	3.25 (0.88,8.03)	5.6 (2.0,12.2)	4.5 (1.7,9.15)	0.97 (0.94, 1.01)	0.116	0.96 (0.92, 0.99)	0.022	1.02 (0.99, 1.05)	0.155			1.05 (1.01, 1.08)	0.009	1.04 (1.01, 1.08)	0.021
Disease severity n (%)															
Old age^a^	102 (73.4)	125 (79.6)	140 (65.4)	1.46 (0.91, 2.33)	0.116			2.07 (1.28, 3.34)	0.003			1.42 (0.83, 2.43)	0.206		
Fever^b^	11 (7.9)	22 (14.0)	20 (9.3)	0.83 (0.39, 1.80)	0.643			1.58 (0.83, 3.01)	0.164			1.90 (0.88, 4.07)	0.1		
Leukocytosis^c^	23 (16.7)	40 (25.5)	31 (14.5)	1.17 (0.65, 2.11)	0.599			2.02 (1.20, 3.41)	0.009			1.72 (0.97, 3.06)	0.063		
**Hypoalbuminemia**^**d**^	25 (18.0)	16 (10.2)	18 (8.4)	2.39 (1.25, 4.57)	0.009			1.24 (0.61, 2.51)	0.558			0.52 (0.26, 1.02)	0.055	0.41 (0.20, 0.84)	0.014
**Azotemia**^**e**^	6 (4.3)	12 (7.6)	5 (2.3)	1.89 (0.56, 6.30)	0.303			3.46 (1.19, 10.03)	0.022	4.06 (1.28, 12.91)	0.018	1.83 (0.67, 5.03)	0.238		
Severity index n (%)															
**Severe CDI**^**f**^	26 (18.7)	48 (30.6)	34 (15.9)	1.22 (0.69, 2.14)	0.492			2.33 (1.42, 3.84)	0.001			1.68 (1.03, 2.72)	0.036	1.88 (1.05, 3.37)	0.034
**Severe CDI**^**g**^	37 (26.6)	55 (35.0)	40 (18.7)	1.58 (0.95, 2.63)	0.079			2.35 (1.46, 3.77)	<0.001	1.87 (1.12, 3.13)	0.016	1.25 (0.93, 1.68)	0.14		
**EIA toxin assay-positive**	90/139 (64.7)	120/156 (76.9)	91/213 (42.7)	2.46 (1.58, 3.83)	<0.001	2.59 (1.65, 4.09)	<0.001	4.47 (2.82, 7.09)	<0.001	4.55 (2.82, 7.33)	<0.001	1.22 (0.64, 2.34)	0.541		
PMC in image study n (%)	13/26 (50.0)	18/41 (43.9)	17/58 (29.3)	2.41 (0.93, 6.26)	0.071			1.89 (0.82, 4.36)	0.137			0.78 (0.29, 2.10)	0.783		
Received treatment n (%)	95 (68.3)	117 (74.5)	114 (53.3)	1.89 (1.21, 2.96)	0.005			2.57 (1.64, 4.02)	<0.001			1.36 (0.82, 2.25)	0.24		
Vancomycin	7 (7.4)	11 (9.4)	10 (8.8)	0.83 (0.30, 2.26)	0.712			1.08 (0.44, 2.65)	0.868			1.31 (0.49, 3.51)	0.598		
Metronidazole	88 (92.6)	106 (90.6)	104 (91.2)												
Clinical outcome n (%)	95	117	114												
Clinical success	72 (75.8)	100 (85.5)	95 (83.3)	0.63 (0.32, 1.24)	0.177			1.18 (0.58, 2.40)	0.655			1.24 (0.71, 2.17)	0.445		
Recurrence^h^	13 (13.7)	20 (17.1)	12 (10.5)	1.54 (0.66, 3.60)	0.324			1.79 (0.82, 3.91)	0.141			1.17 (0.54, 2.54)	0.694		
30-day mortality	13 (9.4)	7 (4.5)	7 (3.3)	3.24 (0.98, 10.67)	0.054			1.23 (0.32, 4.69)	0.764			0.38 (0.13, 1.15)	0.087		
Attributable mortality	3 (2.2)	2 (1.3)	1 (0.5)	3.69 (0.38, 36.02)	0.262			1.97 (0.18, 21.98)	0.583			0.53 (0.09, 3.26)	0.496		

CDI, *Clostridium difficile* infection; WBC, white blood cell; CRP, C-reactive protein; PMC, pseudomembranous colitis; EIA, enzyme immunoassay

^a^Age > 60

^b^Temperature >38.3°C

^c^Peripheral WBC > 15,000/mm3

^d^Serum albumin <2.5 mg/dL

^e^Serum creatinine level ≥ 1.5 times the premorbid level

^f^Leukocytosis or azotemia

^g^Two or more of 4 factors: age > 60 years, temperature > 38.3°C, albumin level < 2.5 mg/dL, or WBC count ≥ 15,000 cells/μL

^h^Recurrence within 30 days after treatment

### Treatment outcomes

Treatment outcomes were made up of clinical success, recurrence rate, 30-day mortality and mortality attributed to CDI, and were determined for all patients receiving CDI treatment for at least 72 hours (Table 2). In univariate analysis, R017 infections caused marginally higher 30-day mortality than the other ribotypes infections (*p* = 0.054), whereas mortality did not differ between R018 and other ribotypes infections or R018 and R017 infections.

### Factors related to 30-day mortality

In order to evaluate factors contributing to 30-day mortality, all the variables including demographics, medical history, clinical variables indicating disease severity, and treatment modalities were compared between surviving CDI patients and those that had died by 30 days post-infection (Table 3). In univariate analysis, death was associated with a higher Charlson’s comorbidity index, receipt of anticancer chemotherapy and infection by the R017 strain, and with leukocytosis, hypoalbuminemia, azotemia and a higher CRP level. In a multiple logistic regression, leukocytosis (OR 2.45, 1.0–6.01, *p* = 0.05) and hypoalbuminemia (OR 4.57, 1.83–11.39, *p* = 0.001) were significantly associated with 30-day mortality, and infection caused by R017 was marginally associated with 30-day mortality (OR 2.14, 0.88–5.22, *p* = 0.094).

**Table 3:** Factors related to 30-day mortality among *Clostridium difficile* patients in a final multiple logistic regression model, 2009–2012

Characteristics	Total patients (n = 510)		simple OR	*p* value	multiple OR	*p* value
	Alive (n = 483)	Expired (n = 27)				
Sex female n (%)	274 (56.7)	14 (51.9)	0.82 (0.38–1.79)	0.619		
Age median (1Q, 3Q)	70 (58,77)	72 (63,78)	1.02 (0.99–1.05)	0.202		
Charlson's score median (1Q, 3Q)	3 (1,5)	5 (2,6)	1.22 (1.06–1.41)	0.006	1.10 (0.93,1.31)	0.281
Hospital days median (1Q, 3Q)	17 (7,42)	19 (7,47)	1 (0.99–1.01)	0.944		
Admission history within 2 months n (%)	172 (35.6)	13 (48.1)	1.68 (0.77–3.65)	0.191		
Medication history n (%)						
Antibiotics use within 2 months	451 (93.4)	27 (100.0)		0.998		
Steroid	139 (28.8)	9 (33.3)	1.24 (0.54–2.82)	0.612		
Chemotherapy	55 (11.4)	8 (29.6)	3.28 (1.37–7.84)	0.008	1.71 (0.58,5.0)	0.328
Immunosuppresants	22 (4.6)	1 (3.7)	0.81 (0.11–6.21)	0.836		
PPI use	180 (37.3)	13 (48.1)	1.56 (0.72–3.4)	0.26		
PCR ribotype n (%)						
Ribotype 017	126 (26.1)	13 (48.1)	2.63 (1.20–5.75)	0.015	2.14 (0.88,5.22)	0.094
Ribotype 018	150 (31.1)	7 (25.9)	0.78 (0.32–1.88)	0.575		
Others	207 (42.9)	7 (25.9)	0.47 (0.19–1.12)	0.089		
Severity factors n (%)						
Old age^a^	345 (71.4)	22 (81.5)	1.76 (0.65–4.74)	0.263		
Fever^b^	50 (10.4)	3 (11.1)	1.08 (0.32–3.72)	0.9		
Leukocytosis^c^	83 (17.2)	11 (40.7)	3.31 (1.48–7.4)	0.003	2.45 (1.0,6.01)	0.05
**Hypoalbuminemia**^**d**^	47 (9.7)	12 (44.4)	7.42 (3.28–16.79)	<0.001	4.57 (1.83,11.39)	0.001
Azotemia^e^	19 (3.9)	4 (14.8)	4.25 (1.34,13.50)	0.014	1.53 (0.38,6.22)	0.555
PMC in image study	45/117 (38.5)	3/8 (37.5)	0.96 (0.22–4.21)	0.957		
CRP median(1Q, 3Q)	4.1 (1.4,9.1)	8 (3.4,15.9)	1.06 (1.02–1.11)	0.006	1.04 (0.99,1.09)	0.115
Treatment n (%)						
Receiving treatment	307 (63.6)	19 (70.4)	1.36 (0.57–3.18)	0.475		
Vancomycin use	26/307 (8.5)	2/19 (10.5)	1.27 (0.28–5.81)	0.757		

PPI, proton pump inhibitor; PMC, pseudomembranous colitis; CRP, C-reactive protein

^a^Age > 60

^b^Temperature >38.3°C

^c^Peripheral WBC > 15,000/mm3

^d^Serum albumin <2.5 mg/dL

^e^Serum creatinine level ≥ 1.5 times the premorbid level

## Discussion

In this study, we assessed the patient demographics, medical conditions, clinical progress and final outcomes of CDI caused by the R017 and R018 strains, the most prevalent strains in Asian countries, compared with those of other ribotypes. In terms of predisposing conditions, R017 infections occurred more frequently in chronically ill patients with higher Charlson’s comorbidity indexes, longer durations of hospitalization, and lower albumin levels. As regards clinical progress, inflammatory markers such as WBC count and serum CRP level were not higher in R017 infections than in infections by other ribotypes, but 30-day mortality was marginally higher. Our finding that R017 infections occurred in more chronically ill patients could explain the marginally higher 30-day mortality. R018 infections occurred in similar patients to infections by other ribotypes but they caused more common severe CDI, and more frequent azotemia despite similar 30-day mortality.

Virulence potential of *C*. *difficile* strains is a combination of different properties such as toxins, adhesion, sporulation and antibiotic resistance, and the major virulence determinants are large clostridial toxins, toxin A (TcdA) and toxin B (TcdB), encoded within the pathogenicity locus (PaLoc) [18]. Although relative pathogenicity of most toxigenic genotype is not clear yet, genetic variants in PaLoc especially in *tcdB* and *tcdC* which is involved in the negative regulation of toxin gene expression may influence the pathogenicity [19]. In a study analyzing sequence variation of *tcdB* and *tcdC* of 1290 clinical isolates from U.K. demonstrate that clade 4 strains to which R017 A-B+ variant strains belong had a relatively homogenous *tcdB* sequences similar to clade 1 strains without *tcdC* variation [19]. Despite relative homogeneous amino acid sequences of TcdB from A-B+ variant *C*. *difficile*, toxin B from variant *C*. *difficile* strain 1470 serotype F (ribotype 017) showed atypical cytopathic effect and differed in protein substrate specificity [20, 21]. As for A+B+ R018 strains, they belonged to clade 1 and had relatively homogeneous *tcdB* sequences with wild type *tcdC* [19]. In terms of virulence factors of *C*. *difficile*, R017 and R018 strains possessed resistance to multiple antibiotics, and impact of *tcdB* sequence variation needs to be further studied as well as other virulence factors.

The fecal toxin A/B enzyme immunoassay (EIA) yields more false negatives in less severe CDI infections [22], and 14-day mortality is higher in EIA-positive patients [23]. Nevertheless, there are many naturally-acquiring *C*. *difficile* strains harboring the *tcdC* mutation without association with severe disease, as well as strains that lack this mutation, but produce copious amounts of toxin (e.g., strain VPI10463) [24, 25]. It is interesting that in the present study the EIA toxin assay was more frequently positive in R018 and R017 infections than in the other infections. Although the final outcome of infections by R018 and R017 was similar to that of the other ribotypes, R018 and R017 infections were treated more commonly than other ribotypes infections and R018 infections caused more severe CDI.

In the light of these findings, we may suppose that the R018 strains produce more toxin or cause more severe infections involving more inflammatory reactions, as shown by the higher positive rate in ELISA toxin assays [23]. With regard to R017 infections, the patients infected by R017 had a marginally higher 30-day mortality rate, which could be explained by greater virulence of R017 represented by higher positive rate of EIA toxin assays or the poorer condition of the patients predisposing them to developing CDIs, or both. The clinical impact of PCR ribotype 027 (R027) strains was debated for several years before studies enrolling large numbers of CDI patients clearly indicated adverse clinical outcomes in such infections [1, 9, 26, 27]. Now, a cohort study including a larger number of CDI patients from multiple centers is needed to establish the clinical outcomes of R017 and R018 infections, and also a study evaluating the virulence of these strains.

R017 and R018 strains are notorious for their high resistance to important antibiotics [9, 28, 29]. It is interesting that exposure to clindamycin, carbapenem and rifampin within the past 2 months was associated with R017 infections, because the R017 isolates were uniformly resistant to clindamycin, and 95% of the R017 strains isolated in our institution were rifampin resistant [30]. Rifampin resistance was previously identified in 5.7% of R018 strains but was not found in other ribotypes [30]. In a European study, rifampin resistance was common in R027, R018 and R356 isolated mainly in Denmark, Hungary, and Italy [28]. The level of rifampin resistance of the prevalent ribotype strains is dependent on their geographic location; all R018 and R356 strains from Italy were of intermediate or full rifampin resistant, but 2 isolates from outside of Italy were less resistant to rifampin [28]. Rifaximin has been used for over 2 decades in Italy, but it was introduced in Korea in 2006 and has not been widely used. Instead, patients infected by R017 were exposed to rifampin before the CDI episode in this study; however it is not clear whether the rifampin resistant R017 strains became resistant to rifampin after exposure to rifampin, or R017 was selected because of pre-existing rifampin resistance of R017 in the patients receiving rifampin. Recently, with the increase of hypervirulent *C*. *difficile* strains, a significant proportion of cases of CDI occurred in the community [31]. These patients were younger and had less severe infection than those with hospital-acquired infection. In Korea, incidence of community acquired CDI does not seem to increase until recently, but it is necessary to observe the trend of CDI occurring in community.

This study had several positive aspects: the number of CDIs caused by R017 and R018 was relatively large compared to other studies [9], and we were able to examine the clinical characteristics of R017 and R018 infections that had high survival fitness in a hospital environment and were suspected of causing serious infections. As for limitations, because the CDI cases and isolates were all from one institution, it is difficult to generalize the results, and the R018 infections did not result in a higher mortality despite yielding more severe clinical findings.

In summary, R018 strains tended to cause azotemia and severe CDI more commonly in the course of infections, and R017 infections occurred more frequently in patients with comorbidities, and resulted more often in hypoalbuminemia. The 30-day mortality was associated with leukocytosis and hypoalbuminemia, and was marginally associated with infection caused by R017.
